# A Novel Biodegradable Tubular Stent Prevents Pancreaticojejunal Anastomotic Stricture

**DOI:** 10.1038/s41598-019-57271-4

**Published:** 2020-01-30

**Authors:** Nader Bakheet, Jung-Hoon Park, Sang Hyun Shin, Sarang Hong, Yejong Park, In Kyong Shim, Changmo Hwang, Jae Yong Jeon, Jorge E. Lopera, Ho-Young Song, Song Cheol Kim

**Affiliations:** 1 Department of Radiology and Research Institute of Radiology, Asan Medical Center, University of Ulsan College of Medicine, 88 Olympic-ro 43-gil, Songpa-gu, Seoul 05505 Republic of Korea; 2 Biomedical Engineering Research Center, Asan Institute for Life Sciences, Asan Medical Center, University of Ulsan College of Medicine, 88 Olympic-ro 43-gil, Songpa-gu, Seoul 05505 Republic of Korea; 3 Department of Surgery, Asan Medical Center, University of Ulsan College of Medicine, 88 Olympic-ro 43-gil, Songpa-gu, Seoul 05505 Republic of Korea; 4 Department of Rehabilitation, Asan Medical Center, University of Ulsan College of Medicine, 88 Olympic-ro 43-gil, Songpa-gu, Seoul 05505 Republic of Korea; 5 Asan Medical Institute of Convergence Science and Technology (AMIST), Asan Medical Center, University of Ulsan College of Medicine, 88 Olympic-ro 43-gil, Songpa-gu, Seoul 05505 Republic of Korea; 6 Department of Surgery, Samsung Medical Center, Sungkyunkwan University School of Medicine, 81 Irwon-ro, Gangnam-gu, Seoul 06351 Republic of Korea; 7 Gastrointestinal Endoscopy and Liver unit, Kasr Al-Ainy, Faculty of Medicine, Cairo University, Cairo, Egypt; 8 Department of Radiology, UT Health Science Center at San Antonio, 7703 Floyd Curl Drive, San Antonio, TX 78229 USA

**Keywords:** Pancreatic cancer, Pancreatic cancer, Preclinical research, Translational research, Biomedical engineering

## Abstract

Stricture of pancreatic-enteric anastomoses is a major late complication of a pancreaticoduodenectomy for the treatment of a periampullary tumor and can lead to exocrine and endocrine insufficiency such as malnutrition and diabetes mellitus. We investigated the safety and efficacy of a biodegradable tubular stent (BTS) for preventing a pancreaticojejunostomy (PJ) anastomotic stricture in both a rat and porcine model. The BTS was manufactured using a terpolymer comprising poly p-dioxanone, trimethylene carbonate, and glycolide. A cohort of 42 rats was randomized into 7 groups of 6 animals each after BTS placement into the duodenum for the biodegradation assay. A total of 12 pigs were randomized equally into a control and BTS placement group. The effectiveness of the BTS was assessed by comparing radiologic images with histologic results. Surgical procedures and/or BTS placements were technically successful in all animals. The median mass losses of the removed BTS samples from the rat duodenum were 2.1, 6.8, 11.2, 19.4, 26.1, and 56.8% at 1, 2, 3, 4, 6, and 8 weeks, respectively. The BTS had completely degraded at 12 weeks in the rats. In the porcine PJ model, the mean luminal diameter and area of the pancreatic duct in the control group was significantly larger than in the BTS group (all *p* < 0.05). BTS placement thus appears to be safe and effective procedure for the prevention of PJ anastomotic stricture. These devices have the potential to be used as a temporary stent placement to treat pancreatic-enteric anastomoses, but further investigations are required for optimization in human.

## Introduction

A pancreaticoduodenectomy (PD) is the standard surgical approach to treating benign and malignant diseases of the pancreatic head and periampullary regions^1–4^. Despite the declining perioperative mortality rate however, surgery-related complications are still frequent in PD cases (30–50%)^4,5^. Most of these adverse events are associated with a pancreaticojejunal (PJ) anastomosis following the PD. Anastomotic leakage and subsequent fistula formation (5–41%), or the later development of anastomotic strictures (5–11%), are the most common complications^3,5–8^. Various modalities have been employed to overcome these adverse events including fibrin sealants^9^, octreotide therapy^10^, varied suturing techniques^11^, and various methods of pancreaticoenteric anastomosis^5^. The current therapeutic strategies remain insufficient however and also somewhat controversial due to inconsistent results from clinical trials and experimental studies^10–13^.

The placement of a trans-anastomotic stent is commonly used to manage a pancreaticoenteric anastomosis^14,15^. Theoretically, the stent would provide protection for the PJ anastomosis by facilitating the precise placement of sutures through the pancreatic parenchyma or duct when performing this procedure. Additionally, it can prevent anastomotic stricture formation at the site of anastomosis late after surgery^16^. Nonetheless, non-biodegradable tubular stent materials (e.g. silicone or polyethylene) may be associated with stent-related complications such as stent migration, obstruction or fracture, stricture of the pancreatic duct, or intestinal obstruction^5,17–20^. Biodegradable stents have the potential to avoid such complications as they spontaneously disappear after healing of the anastomotic site^5,20^. Few studies however have investigated the use of biodegradable stents after PJ anastomosis^21–24^.

We have developed a novel biodegradable tubular stent (BTS) and hypothesized that it may reduce the incidence of anastomotic strictures in patients undergoing PD with PJ anastomosis. The purpose of our present study was to investigate the biodegradable behavior of the BTS in a rat model and further evaluate its safety and efficacy in preventing a PJ anastomotic stricture in a porcine model.

## Materials and Methods

The study were approved by the Institutional Animal Care and Use Committee of Asan Institute for Life Sciences and conformed to US National Institutes of Health guidelines for humane handling of laboratory animals. All animals were housed at one per cage in a room with 12-h light and dark cycles at 24 ± 1 °C with relative humidity of 55 ± 10%. All animals were also acclimatized for at least 1 week before conducting experiments.

### Preparation of a biodegradable tubular stent

The BTS devices used in our study were designed and manufactured using a terpolymer consisting of poly *p*-dioxanone (PDO), trimethylene carbonate (TMC), and glycolide. These stents were made to our specifications by a local manufacturer (S&G biotech, Yongin, Korea) and are not commercially available elsewhere.

**Figure 1:**
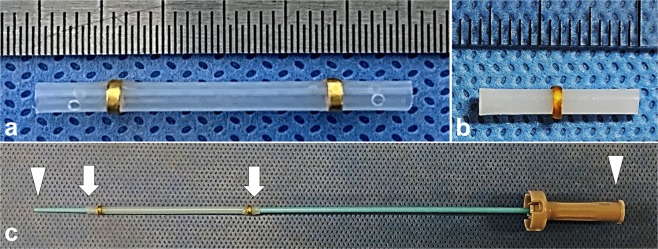
Images of the biodegradable tubular stents (BTSs) used in the rat and porcine animal models. (**a**) A rat BTS (rBTS) with one gold marker at the central portion. (**b**) A porcine BTS (pBTS) with two gold markers and two side holes at each end. (**c**) A 5 Fr dilator (arrowheads) and loaded pBTS (arrows).

The specific BTS used in the rat model (rBTS) was 2 mm in diameter and 15 mm in length. A single gold radiopaque marker was attached to the middle portion of the stent to evaluate its location under fluoroscopy (Fig. 1a). The BTS used in the porcine model (pBTS) was 2 mm in diameter and 30 mm in length. Two gold markers were attached in this case, one at each end, to evaluate the stent location under fluoroscopy and for CT examination. Two side holes were also made at each end of the pBTS to facilitate drainage of the pancreatic secretions and prevent stent obstruction (Fig. 1b). The BTSs were inserted using a 5 Fr dilator (Cook, Bloomington, IN) **(**Fig. 1c).

### BTS placement in the rat

A total of 42 male Sprague-Dawley rats (Orient Bio, Seongnam, Korea) weighing 300–350 g at 10 weeks of age were used for rBTS placement. The rats were randomized after this procedure using computer generated random numbers into seven groups of six rats each. Groups W1, W2, W3, W4, W6, W8, and W12 were sacrificed at 1, 2, 3, 4, 6, 8 and 12 weeks after rBTS placement, respectively.

Prior to stent placement, and after overnight fasting, all rats were anesthetized via an intramuscular injection of a mixture of 50 mg/kg zolazepam and 50 mg/kg tiletamine (Zoletil 50; Virbac, Carros, France) and 10 mg/kg xylazine (Rompun; Bayer HealthCare, Leverkusen, Germany). The anterior abdominal wall hair was shaved, and antisepsis was provided using 0.05% chlorhexidine hydrochloride. A 25 mm midline incision was made in the upper abdomen and the duodenum was identified. After releasing the intestine from the ligament of Trietz, a duodenotomy was performed 60–80 mm from the pylorus, which was chosen to avoid obstruction of the biliopancreatic duct which opens at the medial side of the duodenum (22–28 mm from the pylorus)^25–27^. The rBTS was then placed through the duodenotomy and its distal end was sutured to the duodenal wall using 5-0 non-absorbable sutures (Prolene, Ethicon, Johnson & Johnson Intl, Brussels, Belgium). The duodenotomy was then repaired and the proximal and distal ends of the stent were sutured to the duodenal wall to prevent migration (Supplementary Fig. 1). After all procedures had been completed, the abdominal incision was closed in a layer-by-layer manner. Antibiotics were administered after the operation (0.06 ml of 50 mg/ml ceftriaxone per 100 g of body weight) and continued for 48 hours. Rats in each group were euthanized by administrating inhalable pure carbon dioxide. The body weights of the rats were measured before the procedure and 1, 2, 3, 4, 6, 8 and 12 weeks until sacrifice.

### Biodegradable behavior of the BTS

Each rBTS was removed from the rat duodenum after sacrifice and freeze dried as described previously^28,29^ (Christ freeze dryer type Alpha 1–4 LSC; Martin Christ Gefriertrock-nungsanlagen GmbH, Osterode am Harz, Germany). The surface morphologies of the removed rBTS samples were then assessed using scanning electron microscopy (SEM, Hitachi S4800, Tokyo, Japan) prior to stent placement and at 1, 2, 3, 4, 6, 8 and 12 weeks after rBTS placement. The dried samples were fixed on aluminum pin stubs using a carbon tape, and the surface was analyzed. The weight of the samples was measured using an electronic balance (Ohaus corp., pine Brook, NJ), and the percentage mass loss was calculated as follows: *mass loss (%)* = [*(W* − *W*_*d*_*)/W] × 100*, where W is the original weight of the sample and W_d_ is the weight of dried sample after a certain time interval^30^.

### pBTS placement in a porcine PJ model

A total of 12 pigs weighing 33.7–37.2 kg (median weight = 35.4 kg) were used for pBTS placement. The animals were randomly and evenly divided into two groups using computer generated random numbers as follows: a control group (n = 6) in which PJ was induced but with no stent placement, and a BTS group (n = 6) with induced PJ and a stent placement. All pigs were supplied with food and water *ad libitum* and maintained at 22 ± 2 °C. The body weights of the pigs were measured before surgery and then weekly until sacrifice. The animals were euthanized using an overdose of xylazine hydrochloride (Rompun; Bayer, Seoul, Korea) at 8 weeks after surgery. Histopathological examinations were then performed.

**Figure 2:**
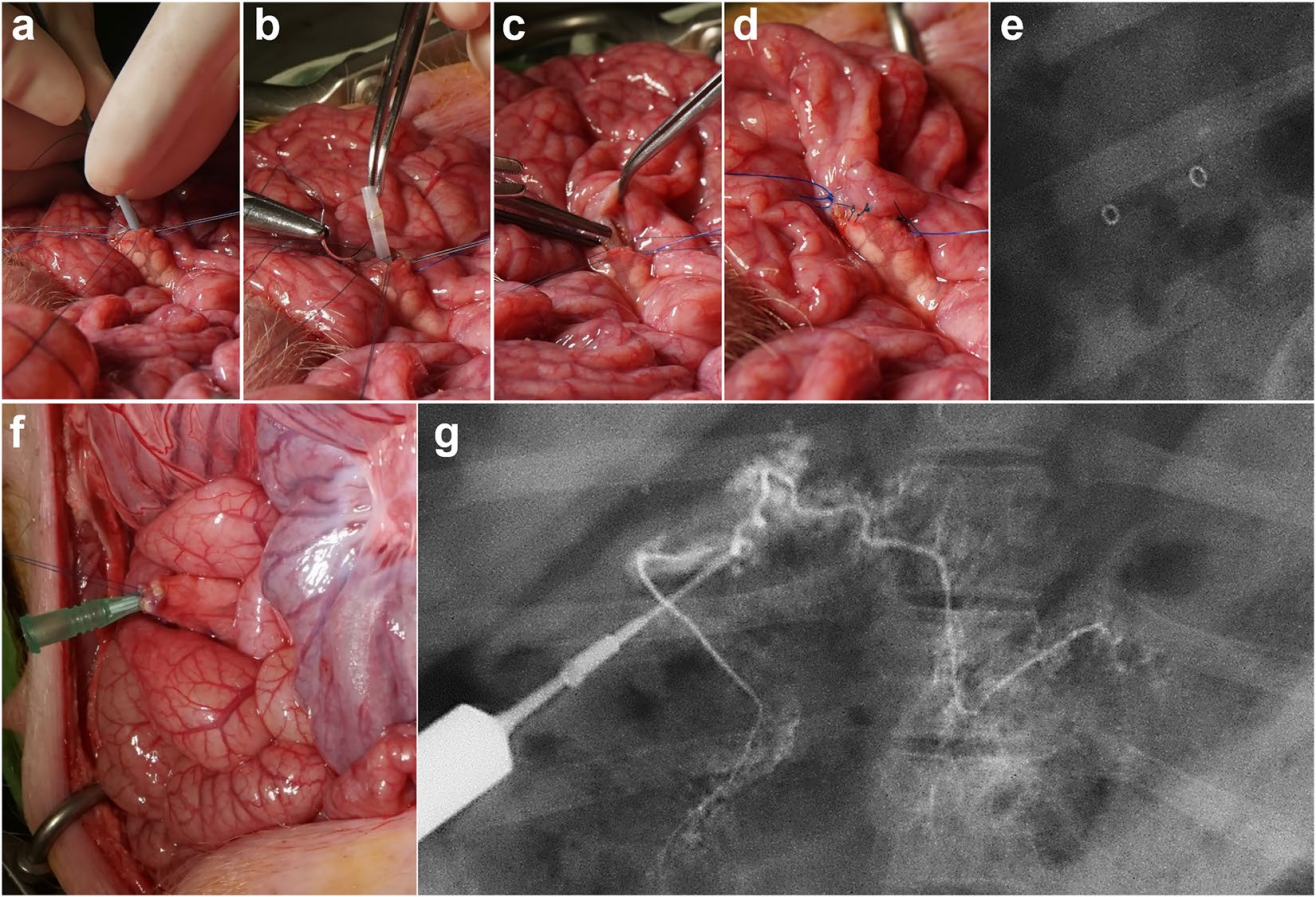
Technical steps in the placement of a pBTS. (**a**) The pBTS with a 5 Fr dilator is inserted into the cut pancreatic duct and (**b**) sutured to the jejunal mucosa to prevent migration. (**c**) The remaining distal portion of the pBTS is inserted into the jejunal loop. (**d**) A pancreatic duct-to-jejunal mucosa anastomosis is successfully performed after pBTS placement. (**e**) Radiograph obtained immediately after the surgical procedure showing the two gold markers of the pBTS. (**f**) An 18 gauge angiocath is inserted into the cut pancreatic duct during the surgery to perform the pancreatic ductography. (**g**) Radiograph of the pancreatic ductography to identify the path of the pancreatic duct. Note. pBTS, porcine biodegradable tubular stent.

The pigs were premedicated with 50 mg intramuscular ketamine after 24 hours of fasting and under the supervision of a veterinarian. An endotracheal tube was then placed, and anesthesia was administered by inhalation of 0.5–2% isoflurane (Ifran®; Hana Pharm. Co., Seoul, Korea) with 1:1 oxygen (510 mL/kg per min). All pigs underwent PJ anastomosis via the duct-to-mucosa method. Following longitudinal abdominal incision in the midline, the duodenum and pancreas were identified. Through tracing along the duodenum, the pancreatic duct was identified at the level of the opening in the ampulla. Following ligation of the pancreatic duct at the duodenal side, the opened duct was anastomosed with an adjacent jejunal loop. Prior to PJ anastomosis, an 18 gauge angiocath (BD Angiocath Plus; BD, Mississauga, Canada) was inserted into the pancreatic duct to perform a pancreatic ductography. Three ml of contrast medium (Omnipaque 300; GE Healthcare, Cork, Ireland) was then injected through the duct to identify the pancreatic duct and to exclude the presence of an accessory pancreatic duct. For the duct-to-mucosa technique, the pancreatic duct-to-jejunal mucosa was stitched with interrupted monofilament synthetic absorbable 5–0 suture material (Monosyn, B. Braun, Melsungen, Germany). In the stent placement group, a pBTS with a 5 Fr dilator was inserted into the cut pancreatic duct, and the sheath was removed with the stent left in place. The remaining distal part of the pBTS was inserted into the jejunal loop. The pBTS was sutured to the jejunal mucosa to prevent stent migration during pancreatic duct-to-jejunal mucosa anastomosis (Fig. 2a–e). A row of non-absorbable continuous stitches made with polypropylene 4-0 sutures was placed in the pancreatic capsule and jejunal serosa in both the anterior and posterior walls of the anastomosis. After all procedures had been completed, the abdominal incision was closed in a layer-by-layer manner. Fluoroscopic images were routinely obtained immediately after the surgical procedure to confirm the location of the pBTS. The body weights of the pigs were measured before and at 1, 4, and 8 weeks after surgery.

#### Pancreatic ductography

An initial pancreatic ductography was performed prior to PJ anastomosis. A follow-up pancreatic ductography was performed immediately after sacrifice to verify the degree of the dilatation in the pancreatic duct and the patency of the anastomotic site (Fig. 2f,g). The luminal diameter was measured with a calibrated catheter (Cook, Bloomington, IN). Photoshop software (version 6.0; Adobe Systems, Palo Alto, CA) was used to acquire digital measurements of the luminal diameter of the pancreatic duct at the anastomotic site, head, upper and lower body, and the tail regions of the pancreas. Measurements were repeated three times at each level, yielding an average value per level, and these values were subsequently averaged to obtain an overall average diameter of the segment. Analyses of the pancreatic ductographic findings were performed on the basis of the consensus of three observers who were blind to the study.

#### Computed tomography (CT)

Abdominal CT (Sensation 16; Siemens, Muenchen, Germany) was performed before and at 1, 4, and 8 weeks after surgery to verify the location of the pBTS, detect any procedure-related complications, and evaluate any changes in the pancreatic duct.

#### Histological analysis

**Figure 3:**
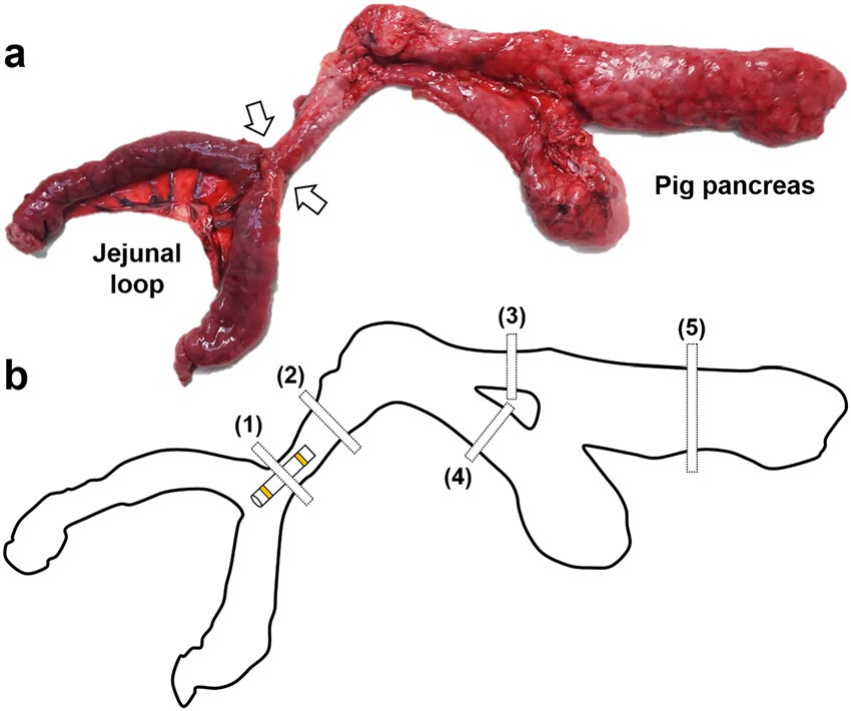
Pathologic findings and locations of the tissues sampled for histologic examination. (**a**) Pancreas, jejunal loop, and pancreaticojejunal anastomosis (arrows) sectioned from a pig immediately after sacrifice. (**b**) Schematic illustration showing the locations of the tissue samples where the pancreatic duct was present at the anastomotic site (1), and at the head (2), upper body (3), lower body (4), and tail (5) portions of the pig pancreas.

Surgical exploration of the pancreas and jejunal loops was followed by a gross examination to evaluate the surgical outcomes with or without stent placement. The pancreas was sectioned transversely at the anastomotic site, head, body, and tail regions for histological analysis to assess any differences between the two groups (Fig. 3). Tissue samples were fixed in 10% neutral buffered formalin for 24 hours and then embedded in paraffin and sectioned. The slides were stained with hematoxylin and eosin and Masson’s Trichrome. The luminal areas of the pancreatic duct at the anastomotic site, head, body, and tail portion were measured to compare the pancreatic duct size between the groups. Histological analysis of the pancreas was performed using a BX51 microscope (Olympus, Tokyo, Japan). Image-Pro Plus software (Media Cybernetics, Silver Spring, MD) was used for these measurements. Histological findings were evaluated based on the consensus of three observers who were blind to the group assignments.

#### Definitions and data analysis

The effectiveness of BTS devices was assessed by technical success rate, incidence of PJ anastomotic stricture, and procedural related complications. Technical success was defined as successful PJ anastomosis with or without pBTS placement. PJ anastomotic stricture was defined as the presence of a fixed narrowing at the anastomotic site, along with distal ductal and side-branch enhancement on pancreatic ductography.

### Statistical analysis

Differences between groups were analyzed using the Mann–Whitney U test as appropriate using SPSS software (version 24.0; SPSS, Inc., Chicago, IL). A *p* value of <0.05 was considered statistically significant.

## Results

### Procedural outcomes in the rat study

**Figure 4:**
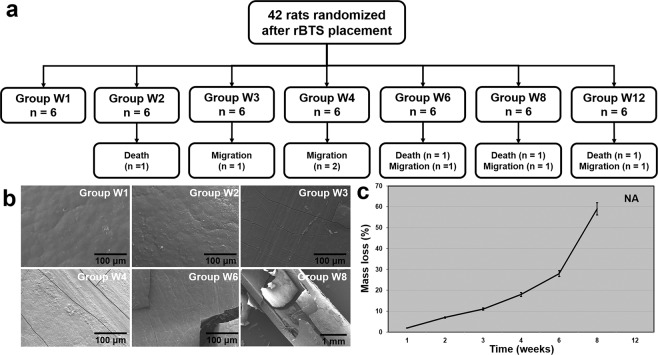
(**a**) Flowchart showing the randomization and the procedural outcomes of the rBTS placement in 42 Sprague-Dawley rats. (**b**) Representative SEM images from group W1 through to group W8. (**c**) Graph indicating the mean percentages of rBTS mass loss. The error-bars represent the standard deviation. Note. rBTS, rat biodegradable tubular stent; SEM, Scanning Electron Microscopy; NA, not applicable.

Placement of the rBTS was technically successful in all rats. Four (9.5%) of the 42 rats died within the first week post-surgery. The autopsies of these animals indicated a leak at the duodenotomy site and peritonitis that was secondary to sepsis, which was the cause of death. Six (15.8%) of the remaining 38 rBTSs were found to have migrated at 3 (n = 1), 4 (n = 2), 6 (n = 1), 8 (n = 1), and 12 (n = 1) weeks after stent placement. The migrated stents in these cases were all eliminated through the anus without any complications. During the fluoroscopic follow-up study, migrated rBTSs were not detected on fluoroscopic images. The 10 rats in which death or stent migration occurred were excluded from further analysis. The remaining 32 (76.2%) rats survived until the end of the study with no stent-related complications (Fig. 4a). There were no differences in body weight changes between the groups (Supplementary Table 1).

### Biodegradable behavior of the stent in the rats

The surface morphology of the removed rBTS revealed minor changes in texture at 1 and 2 weeks, and minor cracks at 3 weeks. After 6 and 8 weeks, small cracks and small pieces partially decomposed from the material were found on the surface of the removed stents (Fig. 4b).

The median mass losses (range) of the removed stents from the rat duodenum were 2.1 (1.2–2.5) %, 6.8 (5.3–8.6) %, 11.2 (9–13.9) %, 19.4 (14.6–20.8) %, 26.1 (25.8–33.9) %, and 56.8 (54.8–56.9) % in groups W1, W2, W3, W4, W6, and W8, respectively (Fig. 4c). In the W12 group, the rBTS had completely decomposed whilst in the rat duodenum and only the sutures used for fixation were remaining.

### Procedural outcomes in the porcine study

**Figure 5:**
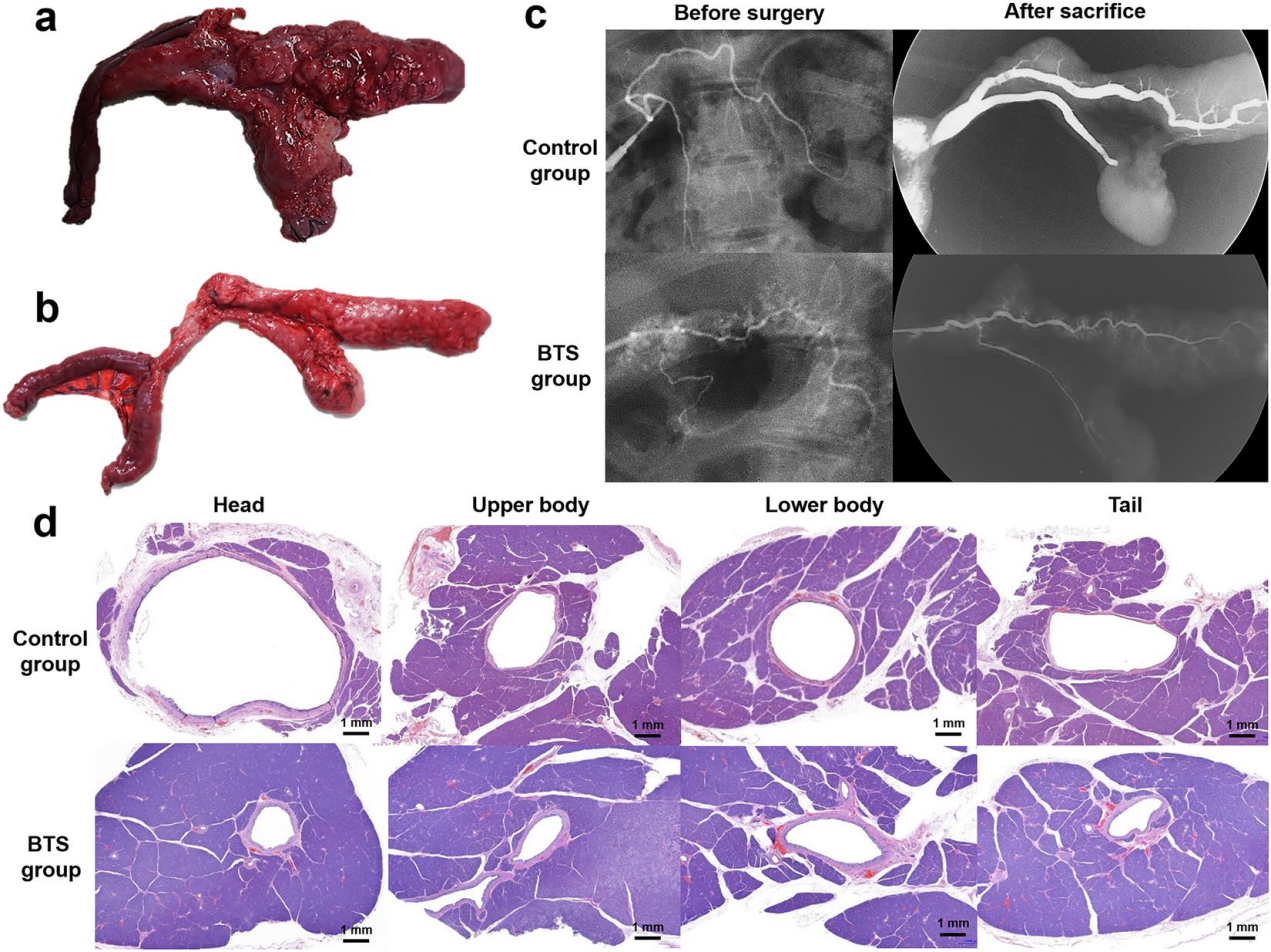
(**a**) Photographs obtained immediately after sacrifice in the porcine study indicating pancreatitis secondary to obstruction at the PJ anastomosis in the control group and (**b**) a normal pancreas in the BTS group. (**c**) Pancreatic ductography images indicating the significant differences in the pancreatic duct luminal diameters between the control and BTS groups. (**d**) Representative hematoxylin and eosin stained images showing the significant differences in the pancreatic duct luminal area between the control and the BTS groups at the head, upper body, lower body and tail portions of the pancreas, respectively. Note. PJ, pancreaticojejunostomy; BTS, biodegradable tubular stent.

The technical success rate of the surgery and pBTS placement in the porcine model was 100%. Two pigs developed an incisional hernia two days after the procedures that were surgically repaired. Post-operative PJ anastomotic stricture occurred in all pigs. However, the degree of stricture and subsequent distal duct dilation in the control group was significantly larger than in the BTS group. Pancreatitis occurred in 2 (16.7%) of the 12 pigs in the control group but in no animals in the BTS group. Severe adhesions, fatty necrosis, and pancreatic tissue changes were observed in an autopsy of the 2 pigs with pancreatitis (Fig. 5a). Two (33.3%) of 6 pBTSs were found to have migrated into the small bowel at 4 weeks after the procedure. There were no differences in the behavior, feeding, and weight changes in the two pigs showing stent migration compared with the remaining 4 pigs. All of the experimental pigs survived until the end of the study (Fig. 5b). There were no significant differences in animal behavior, food intake, and body weight changes between the control and BTS groups (Supplementary Table 2).

#### Pancreatic ductographic findings

The pancreatic ductographic findings in the porcine model are shown in Fig. 5c. The mean luminal diameters of the pancreatic duct at the head (5.65 ± 0.89 mm vs. 1.76 ± 0.46 mm; *p* < 0.001), upper (3.74 ± 1.32 mm vs. 1.58 ± 0.43 mm; *p* = 0.002) and lower (3.59 ± 1.29 mm vs. 1.22 ± 0.67 mm; *p* < 0.001) body, and tail (3.56 ± 1.48 mm vs. 1.25 ± 0.55 mm; *p* = 0.003) regions of the pancreas in the control group were significantly larger than those in the BTS group. However, the mean luminal diameters of the pancreatic duct at the anastomotic site in the control group was significantly smaller than that in the BTS group (0.67 ± 0.43 mm vs. 2.15 ± 0.28 mm; *p* < 0.001). The luminal diameter on pancreatic ductography of the two pigs showing stent migration was also smaller compared to the control group at 8 weeks after surgery (Supplementary Fig. 2c).

#### CT findings

The pBTS was identified to be in place in all of the pigs at the 1 week follow-up CT examination. At 4 weeks however, two of the pBTSs had migrated into the distal small bowel (Supplementary Fig. 2). These migrated stents were eliminated through the anus and could not be visualized at the 8 week follow-up CT examination. No anastomotic leakages or abscess formation occurred in any of the subject pigs during the follow-up study.

#### Histological findings

**Figure 6:**
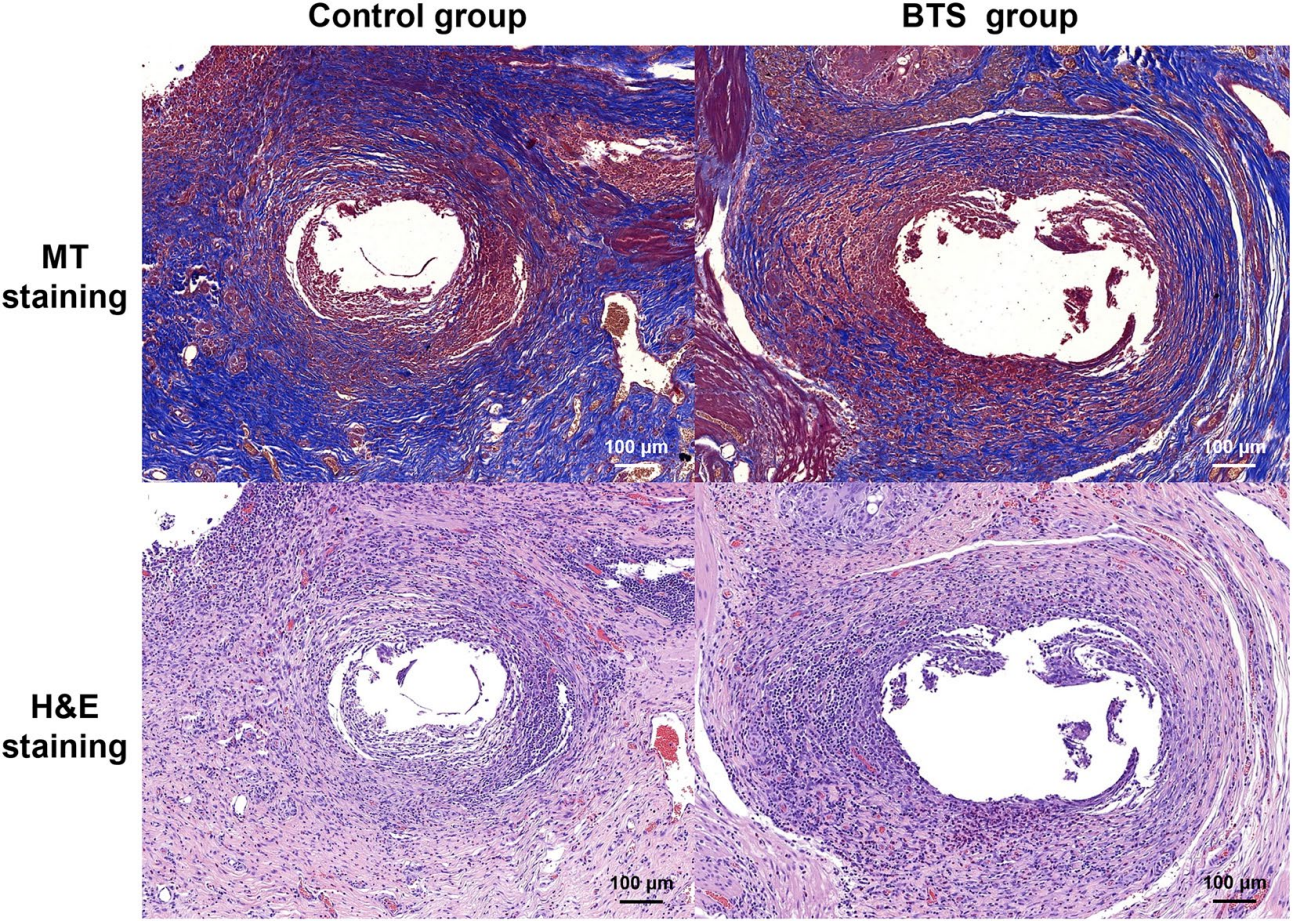
Representative histological images at the PJ anastomotic site indicating a significantly larger luminal area in the BTS group compared to the control group. The extensive fibrotic changes in the control group compared to the BTS group are represented by the blue color of the MT staining. Note. BTS, biodegradable tubular stent; H&E, hematoxylin and eosin; MT, Masson’s trichrome; PJ, pancreaticojejunal.

Histologic findings for the porcine study are shown in Figs. 5d, 6, and Supplementary Fig. 3, and are summarized in Table 1. The mean luminal areas at the head (69.44 ± 52.78 mm^2^ vs. 4.58 ± 2.95 mm^2^), upper (39.43 ± 21.24 mm^2^ vs. 3.96 ± 3.17 mm^2^) and lower (45.74 ± 10.48 mm^2^ vs. 3.60 ± 2.03 mm^2^) body, and tail (23.90 ± 16.82 mm^2^ vs. 3.34 ± 2.05 mm^2^) regions of the pancreas in the control group were significantly larger than the BTS group (all *p* < 0.001). However, the mean luminal area at the anastomotic site in the control group (0.69 ± 0.43 mm^2^) was significantly smaller than that in the BTS group (1.92 ± 0.76 mm^2^) (*p* < 0.001).

**Table 1:** Comparison of pancreatic duct changes at 8 weeks after surgery between the control and BTS groups in the porcine study.

	Anastomosis	Head	Upper body	Lower body	Tail
**Luminal diameters determined by pancreatic ductography (mm)**					
Before surgery (n = 12)	NA	0.87 ± 0.26	0.76 ± 0.22	0.69 ± 0.18	0.41 ± 0.13
Control group (n = 6)	0.67 ± 0.43	5.65 ± 3.89	3.74 ± 2.32	3.59 ± 1.29	3.56 ± 1.48
BTS group (n = 6)	2.15 ± 0.28	1.79 ± 0.86	1.58 ± 1.12	1.22 ± 0.67	1.25 ± 0.55
*p*-valve*	<0.001	<0.001	0.002	<0.001	0.003
**Luminal areas determined by histological findings (mm**^**2**^**)**					
Control group (n = 6)	0.69 ± 0.43	69.44 ± 52.78	39.43 ± 21.24	15.74 ± 10.48	23.90 ± 16.82
BTS group (n = 6)	1.92 ± 0.76	4.58 ± 2.95	3.96 ± 3.17	3.60 ± 2.03	3.34 ± 2.05
*p* = valve*	<0.001	<0.001	<0.001	<0.001	<0.001

Note. Data values are the mean ± standard deviation (mm). BTS, biodegradable tubular stent; NA, not applicable

*Comparison between control and BTS groups, Mann-Whitney t test.

## Discussion

A novel BTS from commercially available materials was successfully fabricated to reduce the incidence of complications in patients with pancreatic-enteric anastomoses. The results of the present animal studies suggest that a temporary BTS placement effectively prevents PJ anastomotic strictures. Our current results in the rat model demonstrated an initial degradation at 3 weeks after placement, which was complete between 8 and 12 weeks without stent-related complications. In our porcine model study, the luminal diameter of the pancreatic duct was significantly smaller in the BTS group than in the control group, indicating effective prevention of anastomotic stricture formation. Consistent with these gross findings, histologic results revealed a significantly reduced luminal area in the BTS group, which correlated with the pancreatic ductography findings. The stents migrated into the small bowel at between 1 and 4 weeks after surgery in 2 of 12 pigs. Nonetheless, all of the pigs in the BTS group showed a patent anastomotic site, and the pancreatic duct diameter was significantly smaller than that in the control group.

The incidence of PJ anastomotic stricture after PD is increasingly being recognized due to the greater repertoire of surgical indications, increased safety of modern procedures and improved patient survival after these surgeries. The use of a transanastomotic pancreatic stent after PJ anastomosis to reduce the risk of post-operative complications is still controversial. Some studies have found no benefits and even complications after using either internal or external stents, while others reports have indicated that stenting may decrease the risk of complications^5,8,14–16,19^. Surgeons who recommend the transanastomotic stent believe that it can maintain anastomotic patency and thus prevent adverse outcomes such as edema-induced postoperative pancreatitis and/or partial dehiscence of the PJ anastomosis. Additionally, the use of a stent can prevent late complications such as a stricture formation at the pancreatic duct, and facilitate the long-term patency of the anastomosis, thereby helping to avoid secondary pancreatic fibrosis, atrophy, or exocrine dysfunction^5^. In our current porcine model, the placed pBTS maintained the patency of the PJ anastomosis in all of the experimental pigs during a 12 week follow-up. Moreover, the luminal diameters and areas of the pancreatic duct were significantly less dilated in the BTS group compared with the control group of these animals. This was also the case in the 2 pigs that showed stent migration into the small bowel, thus indicating that the BTS prevented stricture at the anastomotic site even with a short retention time. Furthermore, 2 pigs in the control group developed pancreatitis, but no such adverse events occurred in the BTS group. This may be explained by the marked narrowing of the PJ anastomotic site in the control group, as detected by the pancreatic ductography, which could have resulted in reflux of the pancreatic secretions and autodigestion of the pancreatic tissue by pancreatic enzymes, followed by secondary pancreatitis. There was no evidence that this occurred in the BTS group, likely because the patent anastomotic site allowed adequate drainage of pancreatic secretions in these animals. Pancreatitis did not occur in our rat study as the purpose of these experiments was to evaluate the time needed for the BTS to degrade when exposed to bile, pancreatic, and intestinal secretions. Hence, the rBTSs were placed into the third portion of the rat duodenum beyond the opening of the biliopancreatic duct and not into the pancreatic duct. Kasuya *et al*.^23^ have previously investigated a novel anastomosis technique involving a short-term degradable stent in PJ anastomosis cases. That stent was made from monocryl and PDS-II and was evaluated in 8 patients with PJ after PD. It preserved its strength for about 3 weeks and few post-operative complications were reported. Nordback *et al*.^30^ investigated a radiopaque biodegradable stent in human phase I trial. No in-hospital mortalities was reported in that study and the authors observed an overall fistula rate of 3%, and an overall hemorrhage rate of 7%. Most of the patients however had a well opened (57%), or slightly narrowed (13%) anastomotic site.

The optimal duration of stent placement in PJ anastomosis is still the subject of debate^31^. Notably however, there are limited data in support of the long-term use of pancreatic stents, and some studies have suggested that long-term transanastomotic stent placement is of no benefit and can even be harmful^5^. Biodegradable materials vary in their degradation time and whilst some can degrade in only a few weeks, others can take up to several months or even years^21,32^. Unpublished data from our own *in-vitro* experiments has indicated that the BTS starts to degrade at around 20 days when placed in pancreatic or bile juice. In our current rat experiments, the BTS stent started to show signs of degradation after 3 weeks and had completely degraded after 12 weeks. The results from our present porcine model analysis indicated complete healing of the anastomosis with no stricture or obstruction in the pancreatic duct in the BTS group. This benefit was still evident in 2 of the pigs even after the stent had migrated before the completion of the study period. This indicated that pBTS had remained in place for a sufficient time for the anastomotic site to heal.

Mari *et al*.^18^ have previously reviewed studies describing stent-related complications after PJ. Stent retention was reported in six prior studies and the diagnosis was made after a variable duration from 6 weeks to up to 7 years after surgery. Stent retention usually resulted in steatorrhea, pancreatitis, weight loss, or abdominal pain and endoscopic or surgical removal of the stents was required in all cases. In that same review, 7 previous studies were described that had reported stent migration. The time range to diagnosis was even wider in these cases than in the patients with stent retention (from several months to 19 years). Stent migrations resulted in a range of complications including small bowel obstruction or perforation, bezoar formations, dehiscence of the PJ anastomosis with migration to the peritoneal cavity with peritonitis, liver abscess, and cholangitis. Endoscopic or surgical intervention was needed in most of these cases.

In our current porcine study, there were no instances of pBTS retention, likely due to the relatively rapid degradation of this stent. However, although the pBTS in each animal was sutured to the jejunum during the surgery, migration into the small bowel occurred in 2 of 6 pigs. Degradation of this stent at the site of the suture attachment is thought to be the reason for this. The migrated pBTS was easily detected on fluoroscopy and/or CT examination due to attached gold markers in the small bowel during the follow-up studies. The migrated pBTSs passed through the anus in both animals but the relatively short duration to the complete degradation of these stents (less than 12 weeks) made it unlikely that we would see any future sequelae. Other possible non-biodegradable stent-related complications that have been described previously include stent obstruction and later stricture of the pancreatic duct secondary to an inflammatory reaction to the stent. None of these adverse outcomes was encountered in our present study mostly due to the short stent-retention time.

The BTS has several advantages over conventional stents for pancreatic-enteric anastomoses. First, it is easy and safe to place the BTS because of its tapered introducer (5 Fr in diameter). Second, the side holes at each end of the BTS may have assisted with the drainage of pancreatic secretions and decreased the risk of obstruction. Third, the location of the placed BTS in the pancreatic-enteric anastomoses can be easily evaluated through a radiological examination because of the gold markers. A further important advantage of the BTS is that removal is not needed due to its biodegradable features, making it a potentially safer therapeutic option.

This study had some limitations of note. First, although many of the variables of interest reached statistical significance, the number of experimental animals was too small to perform a robust statistical analysis. Second, physical stent changes (e.g. changes in tensile strength) were not measured. Lastly, the results of the pancreatic ductography could be intrinsically inaccurate. This is because the luminal diameter of the pancreatic duct and quantification of the tissue response could differ due to the degree of manual injection of contrast medium. Nevertheless, although additional studies are required to verify the efficacy and safety of the BTS in comparison studies, our current findings provide a basic proof of concept for its use to prevent PJ anastomotic stricture after surgery. Our present results have thus yielded important insights into the potential benefits of a biodegradable stent placement in the management of PJ after PD.

In conclusion, BTS placement appears to be safe and effective procedure for the prevention of a PJ anastomotic stricture. These devices therefore have the potential to be used as a temporary stent placement to treat pancreatic-enteric anastomoses, but further investigations are required for optimization in human.

## Supplementary information

Supplementary Information.
