# Supplementary material for: A Novel Biodegradable Tubular Stent Prevents Pancreaticojejunal Anastomotic Stricture
Source: Sci Rep. 2020 Jan 30;10:1518. doi: 10.1038/s41598-019-57271-4 (PMC6992790; doi:10.1038/s41598-019-57271-4)
Supplement: Supplementary file 1 — Supplementary Information. [file 41598_2019_57271_MOESM1_ESM.docx]

**SUPPLEMENTAL INFORMATION**

**A Novel Biodegradable Tubular Stent Prevents Pancreaticojejunal Anastomotic Stricture**

Nader Bakheet, M.D., Ph.D.^1, 7*,^ Jung-Hoon Park, Ph.D.^1,2*^, Sang Hyun Shin, M.D., Ph.D.^3,6*^, Sarang Hong, M.D.^3^, Yejong Park, M.D.^3^, In Kyong Shim, Ph.D.^2^, Changmo Hwang, Ph.D.^2^, Jae Yong Jeon, M.D.^4^, Jorge E. Lopera, M.D.^8^, Ho-Young Song, M.D., Ph.D.^1+^, Song Cheol Kim, M.D., Ph.D.^2,3,5+^

*Departments of ^1^Radiology and Research Institute of Radiology, ^2^Biomedical Engineering Research Center, Asan Institute for Life Sciences, ^3^Surgery, ^4^Rehabilitation, and ^5^Asan Medical Institute of Convergence Science and Technology(AMIST), Asan Medical Center, University of Ulsan College of Medicine, 88 Olympic-ro 43-gil, Songpa-gu, Seoul, 05505, Republic of Korea; ^6^Department of Surgery, Samsung Medical Center, Sungkyunkwan University School of Medicine, 81 Irwon-ro, Gangnam-gu, Seoul, 06351, Republic of Korea; ^7^Gastrointestinal Endoscopy and Liver unit, Kasr Al-Ainy, Faculty of Medicine, Cairo University, Cairo, Egypt. ^8^Department of Radiology, UT Health Science Center at San Antonio, 7703 Floyd Curl Drive, San Antonio, TX, 78229, USA*

* N.B., J.-H.P. and S.H.S. contributed equally to this work and are the co-first authors.

+ H.-Y.S. and S.C.K. contributed equally to this work and are the co-corresponding authors.

**Correspondence:**

Ho-Young Song, M.D., Ph.D.^1^ and Song Cheol Kim, M.D., Ph.D.^2^

^1^Department of Radiology, Asan Medical Center, University of Ulsan College of Medicine, 88 Olympic-ro 43-gil, Songpa-gu, Seoul 05505, Republic of Korea

Tel: 82-2-3010-4370 Fax: 82-2-476-0090

E-mail: [hysong@amc.seoul.kr](mailto:hysong@amc.seoul.kr)

^2^Division of HBP Surgery, Department of Surgery, Asan Medical Institute of Convergence Science and Technology(AMIST), Asan Medical Center, University of Ulsan College of Medicine, 88 Olympic-ro 43-gil, Songpa-gu, Seoul 05505, Republic of Korea

Tel: 82-2-3010-3936 Fax: 82-2-476-0090

E-mail: drksc[@amc.seoul.kr](mailto:hyjung@amc.seoul.kr)

**
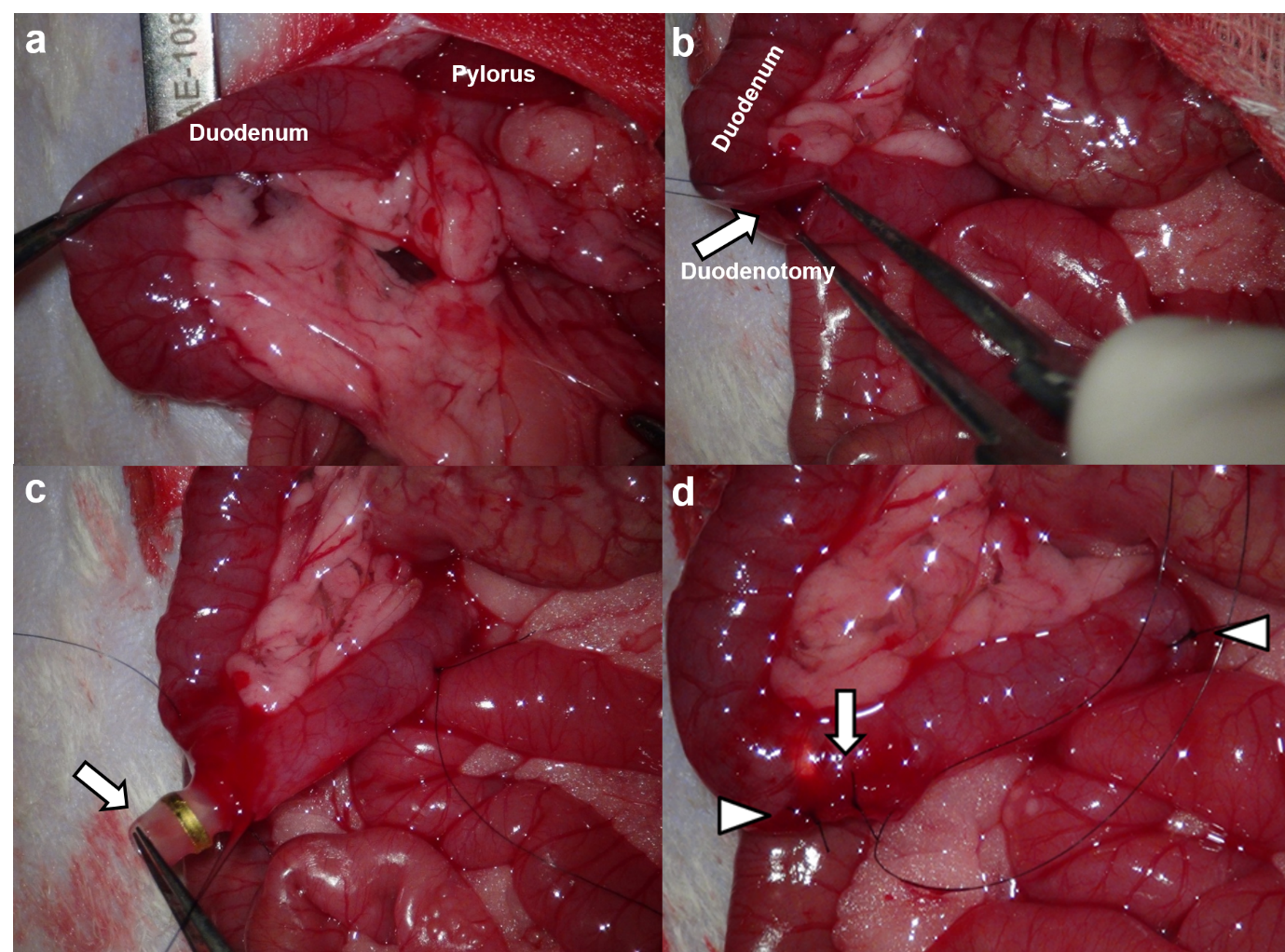
**

**Supplementary Figure 1.** Technical steps in the placement of an rBTS. (a) Identifying the duodenum and pylorus. (b) A duodenostomy (arrow) is performed at approximately 6 to 8 cm distal from the pylorus. (c) The rBTS (arrow) is placed through the duodenostomy. (d) The duodenostomy is closed (arrow), and both ends of the rBTS (arrowheads) are sutured to the duodenal wall.

Note. rBTS, rat biodegradable tubular stent

**Supplementary Table 1.** Effects of rBTS placement on body weight changes in the rats

| **Groups** | **Body weight (g, mean ± SD)** | | | | | | |
| --- | --- | --- | --- | --- | --- | --- | --- |
|  | **Before** | **1 week** | **2 weeks** | **4 weeks** | **6 weeks** | **8 weeks** | **12 weels** |
| W1 | 285.2 ± 20.2 | 310.3 ± 19.3 | - | - | - | - | - |
| W2 | 274.4 ± 22.1 | 315.2 ± 21.3 | 339.8 ± 22.1 | - | - | - | - |
| W3 | 286.1 ± 29.6 | 324.5 ± 22.1 | 361.5 ± 20.2 | - | - |  | - |
| W4 | 297.8 ± 23.2 | 353.1 ± 22.4 | 382.2 ± 21.4 | 402.4 ± 20.1 | - | - | - |
| W6 | 283.7 ± 25.5 | 324.8 ± 24.1 | 369.3 ± 26.3 | 410.4 ± 21.6 | 440.6 ± 24.1 | - | - |
| W8 | 275.2 ± 25.1 | 305.9 ± 23.7 | 350.7 ± 24.2 | 393.9 ± 21.4 | 435.4 ± 22.6 | 483.4 ± 25.2 | - |
| W12 | 302.3 ± 25.1 | 329.2 ± 19.1 | 379.6 ± 19.2 | 405.6 ± 22.5 | 445.2 ± 20.1 | 482.7 ± 21.6 | 502.3 ± 22.5 |

Note. rBTS, rat biodegradable tubular stent; SD, standard deviation.

**Supplementary Table 2.** Effects of pBTS placement on body weight changes in the pigs

| **Groups** | **Body weight (kg, mean ± SD)** | | | | **Weight change (%)** |
| --- | --- | --- | --- | --- | --- |
|  | **Before** | **1 week** | **4 weeks** | **8 weeks** |  |
| Control | 35.9 **±** 3.9 | 35.6 **±** 4.5 | 41.2 **±** 4.7 | 48.7 **±** 5.5 | 73.7 % |
| BTS | 34.3 **±** 4.4 | 35.5 **±** 5.2 | 42.7 **±** 5.6 | 47.8 **±** 7.9 | 71.8 % |

Note. pBTS, porcine biodegradable tubular stent; SD, standard deviation.


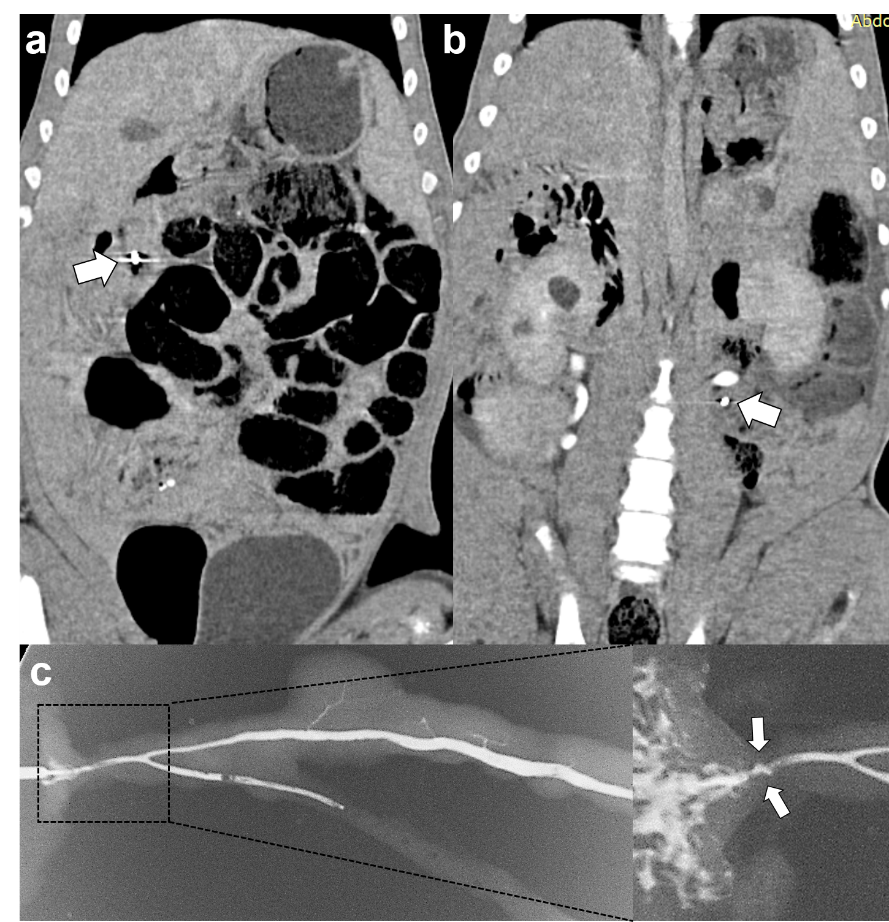


**Supplementary Figure 2.** CT images showing pBTS migration into the small bowel in the BTS group of pigs. (a) CT coronal image obtained at 1 week after pBTS placement showing the gold marker of this stent at the PJ anastomotic site. (b) Follow-up CT image obtained 4 weeks after pBTS placement indicating stent migration into the small bowel. (c) Pancreatic ductography obtained immediately after sacrifice showing good patency of the contrast medium through the anastomotic site (arrows) and the slightly dilated pancreatic duct.


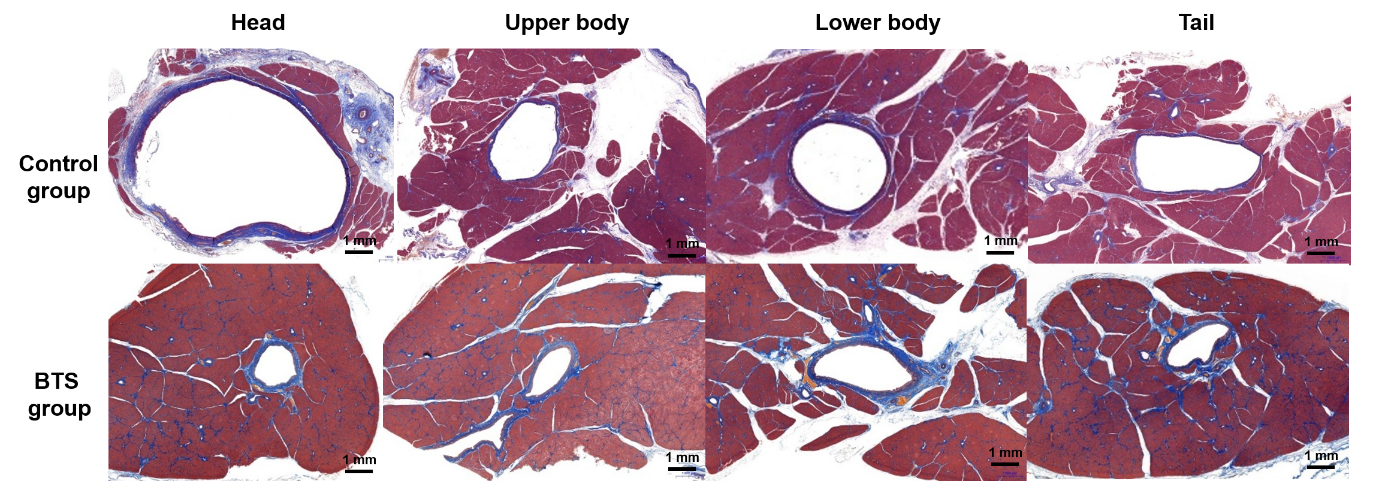


**Supplementary Figure 3.** Representative Masson’s Trichrome stained images showing the significant differences in the pancreatic duct luminal area between the control and the BTS groups.
